# Supplementary material for: Rescue of Mitochondrial SIRT3 Ameliorates Ischemia-like Injury in Human Endothelial Cells
Source: Int J Mol Sci. 2022 Aug 14;23(16):9118. doi: 10.3390/ijms23169118 (PMC9409423; doi:10.3390/ijms23169118)
Supplement: Supplementary file 1 [file ijms-23-09118-s001.zip › ijms-1818583-supplementary.pdf]

International Journal of
Molecular Sciences

MDPI

Article

Rescue of Mitochondrial SIRT3 Ameliorates Ischemia-like Injury in Human Endothelial Cells

Xi Liu [1], Yi Li [1], Zhen Zhang [1], Juan Lu [1], Gang Pei [1,2,3,*] and Shichao Huang [1,*]

1 State Key Laboratory of Cell Biology, Shanghai Institute of Biochemistry and Cell Biology, Center for Excellence in Molecular Cell Science, Chinese Academy of Sciences, Shanghai 200031, China
2 Shanghai Key Laboratory of Signaling and Disease Research, Laboratory of Receptor-Based Biomedicine, The Collaborative Innovation Center for Brain Science, School of Life Sciences and Technology, Tongji University, Shanghai 200070, China
3 Institute for Stem Cell and Regeneration, Chinese Academy of Sciences, Beijing 100045, China
* Correspondence: gpei@sibs.ac.cn (G.P.); huangshichao@sibcb.ac.cn (S.H.)

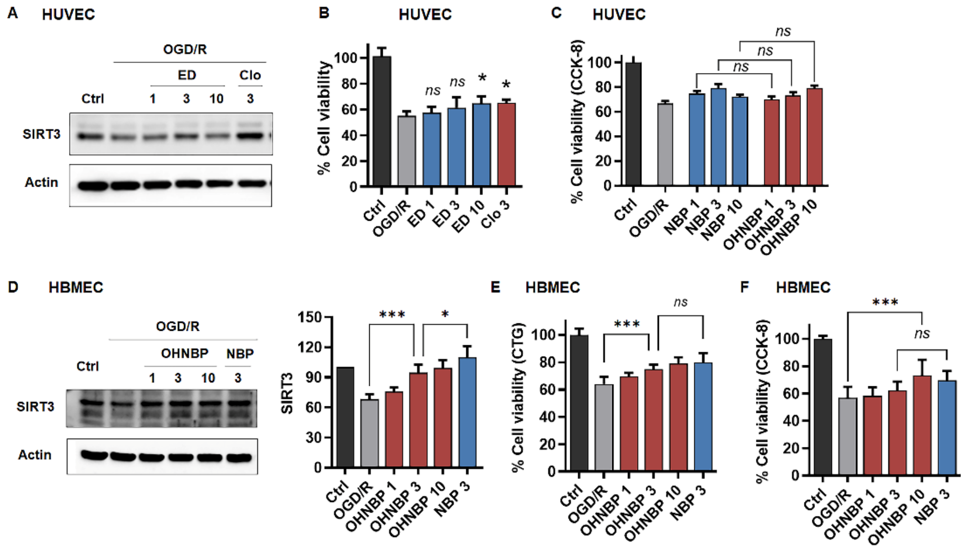

Figure S1. Clinical drugs rescue SIRT3 to reduce OGD/R-induced injury. (A) The effect of ED on SIRT3 expression in HUVECs after exposure to OGD/R as assessed by Western Blotting. (B) The effect of ED on cell viability of HUVECs after exposure to OGD/R by using CTG assay. (C) The effect of OHNBP and NBP on cell viability in HUVECs after exposure to OGD/R by using CCK-8 assay. (D) The effect of OHNBP and NBP on SIRT3 expression in HBMECs after exposure to OGD/R as assessed by Western Blotting. (E-F) The effect of OHNBP and NBP on cell viability in HBMECs after exposure to OGD/R by using CTG assay, or CCK-8 assay. The data are presented as the means ± SD, and were assessed using one-way ANOVA. * $p < 0.05$ vs. the OGD/R group, *** $p < 0.001$ vs. the OGD/R group. ns, no significance, n = 3 for all.

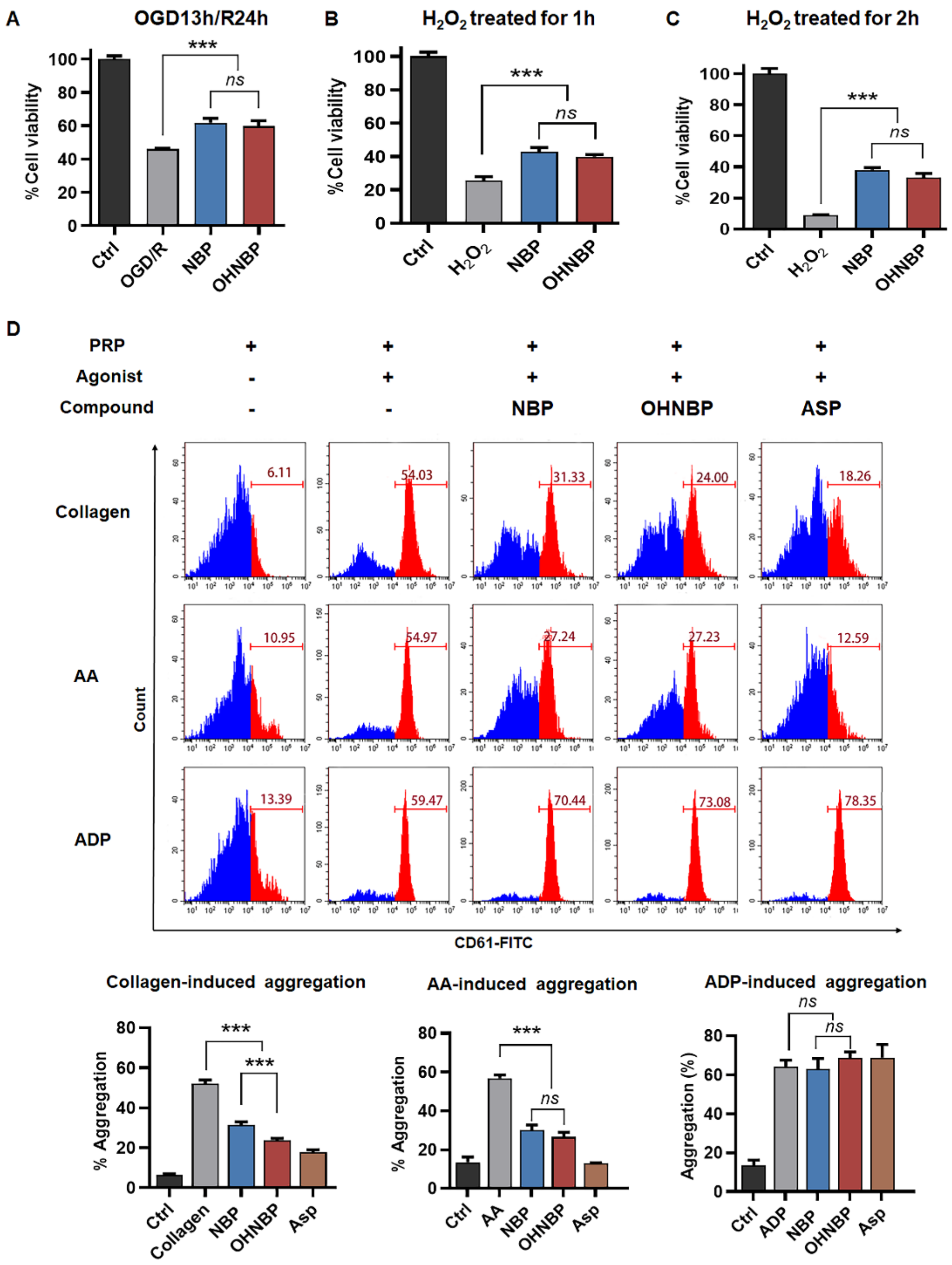

Figure S2. OHNBP has better pharmacology effect than NBP in neuroprotection, anti-oxidation, and anti-aggregation. (A) The effect of OHNBP or NBP on cell viability of SK-N-SH cells induced by OGD13.5h/R24h. (B-C) The effect of OHNBP or NBP on cell viability of SK-N-SH cells damaged by 1 mM H2O2 for 1 h or 2 h. (D) The effect of OHNBP, NBP, or Asp on platelet aggregation induced by 10 μg/mL collagen, 1 mM AA, or 10 μM ADP. The data are presented as the means ± SD, and were assessed using one-way ANOVA. *** $p < 0.001$. ns, no significance, n = 3 for all.
